# The Expression of F2RL1, P2RX2, P2RX3 and P2RY2 in the Esophagus of Patients with Gastroesophageal Reflux Disease and Their Relationship to Reflux Symptoms—A Pilot Study

**DOI:** 10.3390/jcm14061884

**Published:** 2025-03-11

**Authors:** Anna Mokrowiecka, Adrian Bartoszek, Adam Fabisiak, Agata Wróbel, Jakub Fichna, Agnieszka Wierzchniewska-Ławska, Damian Jacenik, Ewa Małecka-Wojciesko

**Affiliations:** 1 Department of Digestive Tract Diseases, Faculty of Medicine, Medical University of Lodz, 90-153 Lodz, Poland; 2 Department of Biochemistry, Faculty of Medicine, Medical University of Lodz, 92-215 Lodz, Poland; 3 Department of Pathology, Faculty of Medicine, Medical University of Lodz, 92-213 Lodz, Poland; 4 Department of Cytobiochemistry, Faculty of Biology and Environmental Protection, University of Lodz, 90-236 Lodz, Poland

**Keywords:** F2RL1, GERD, PAR, P2RX2, P2X2, P2RX3, P2RY2

## Abstract

**Background:** The current treatment of gastroesophageal reflux disease (GERD) is focused on decreasing gastric acid secretion. However, there is still a group of patients that do not respond to conventional therapy. Proteinase-activated receptors and purinergic receptors have been implicated in inflammation, visceral hyperalgesia and esophageal hypersensitivity. The aim of this study was to evaluate the esophageal expression of PAR2 (*F2RL1*) and P2RX2, P2RX3 and P2RY2 in GERD patients. **Methods:** A total of 53 patients with GERD and 9 healthy controls were enrolled in this study. The expression of the studied receptors was quantified using real-time PCR on esophageal biopsies from the patients with GERD and healthy controls. The correlation between the dilated intracellular spaces (DIS) score and patients’ quality of life was investigated. **Results:** PAR2 receptor expression was higher in ERD compared to NERD and controls (326.10 ± 112.30 vs. 266.90 ± 84.76 vs. 77.60 ± 28.50; NS). P2X2 exhibited the highest expression in NERD compared to ERD and controls (302.20 ± 82.94 vs. 40.18 ± 17.78 vs. 26.81 ± 10.27), similarly to P2Y2, which expression was higher in NERD than in ERD and controls (7321.00 ± 1651.00 vs. 5306.0 ± 1738.00 vs. 3476.00 ± 508.0). **Conclusions:** We found that the expression of *F2RL1*, *P2RX2* and *P2RY2* is positively correlated to the DIS score in GERD patients. Higher PAR2, P2X2 and P2Y2 expression could mediate the sensitization of the esophagus and may be associated with the higher intensity of symptoms perceived by NERD patients.

## 1. Introduction

Gastroesophageal reflux disease (GERD) is a chronic condition caused by irritation of the esophageal lining due to regurgitation of acidic stomach contents [1]. GERD affects approximately 2.5% of the adult population in East Asia and nearly 25% of the adult population in North America and Europe [2,3]. The molecular basis of GERD is complex and involves esophageal motor abnormalities, visceral hypersensitivity, impaired mucosal resistance and signs of esophageal inflammation [3,4,5]. The current treatment of GERD relies on the administration of proton-pump inhibitors (PPIs) to reduce gastric acid secretion. However, a subset of patients does not respond to conventional therapy, and novel therapeutic approaches are needed [3,6].

Non-erosive reflux disease (NERD), which accounts for approximately 70% of GERD cases, is primarily characterized by typical gastroesophageal reflux symptoms in the absence of macroscopic damage or visible inflammation on endoscopy [3]. A poor response to PPIs and a high recurrence rate are key features of NERD [7].

It was found that, besides acid, also bile, pancreatic trypsin or tryptase, are components of the refluxate [8]. On exposure to the weakly acidic solutions, the impairment in integrity is greatest in symptomatic NERD patients. This concerns particularly patients with poor response to PPIs (refluxate is most often weakly acidic in these cases). In patients with NERD, it seems that the mucosal integrity is more vulnerable to the acid exposure [8]. Impairment of esophageal mucosal integrity caused by refluxate increased the access of noxious components through the epithelial barrier to areas of dense nociceptor presence and led to a state of esophageal sensitization and to symptoms occurrence in NERD [8]. A deeper understanding of the mechanisms underlying NERD is crucial for developing new therapeutic strategies to improve clinical outcomes in refractory reflux diseases [4,9].

Protease-activated receptors and purinergic receptors (also known as purinoceptors) are widely expressed in human tissues and appear to regulate numerous processes, such as visceral pain, motility and immune response [9,10,11,12]. Protease-activated receptor 2 (PAR2, encoded by the F2RL1 gene [coagulation factor II thrombin receptor-like trypsin receptor 1]) is specifically activated by serine proteases, including trypsin and mast cell-derived tryptase and belongs to the 7-transmembrane G-protein-coupled receptor family [8]. In esophageal squamous cell lines, PAR2 expression is induced by exposure to acid and weakly acidic solutions [1]. When activated by trypsin in refluxate, the trypsin-PAR2 receptor complex mediates relaxation in the lower esophageal sphincter (LES) in guinea pigs [13,14]. Furthermore, PAR2 induces both proinflammatory and neuroinflammatory effects [14,15]. NFkB- and AP-1-dependent IL-8 upregulation following PAR2 stimulation by trypsin appears to be the mechanism underlying esophageal inflammation when the distal esophagus is exposed to duodenal reflux containing trypsin [9].

Purinergic receptors are membrane-bound receptors that use nucleoside tri- and diphosphates such as ATP, UTP, ADP and UDP or adenosine as transmitters [5]. There are three major subfamilies, P1, P2X and P2Y, which exert their effect through either ligand-gated ion channels (P2X) or by being G protein–coupled receptors (P1 and P2Y) [5]. The significance of purinergic receptors in the pathogenesis of GERD is still unknown. Animal studies have provided evidence of purinergic upregulation in mucosal tissue and/or afferent nerves in both GERD and inflammatory bowel disease [16]. Although no clear evidence supports neural purinergic upregulation in GERD in humans, purinergic receptors may contribute to GERD-related symptoms by enhancing esophageal nociception and hypersensitivity. Thus, they could represent a potential target for future pharmacological treatments of [5]. Nevertheless, a limited number of studies have examined the potential role of protease-activated and purinergic receptors in GERD.

Here, we hypothesize that the expression of PAR_2_ and selected purinoceptors from P2X and P2Y family may be associated with GERD clinical manifestation. Therefore, we compared the levels of the studied receptors in various groups of patients, including patients with NERD, depending on the microscopic changes of the esophagus and dilated intercellular spaces (DIS) assessment. An association has been found between DIS and exposure to acid, acid–pepsin, bile and stress. DIS appears to be associated with symptoms of reflux, even more than other histologic parameters, and disappears with resolution of symptoms after treatment [3,17,18]. DIS in basal and suprabasal areas gives the refluxate access to the chemosensitive nerves found in the deep layers of the esophageal squamous mucosa. These chemosensitive nerves can then express and activate PAR2, causing symptoms [3,17,18,19].

Moreover, we also assessed *F2RL1*, *P2RX2*, *P2RY2* and *P2RX3* expression in correlation with symptoms affecting patients’ quality of life, using a GERD- HRQL questionnaire [20]. This is a symptoms-based questionnaire, which allows assessing the severity of the disease from the patient’s perspective. Using this questionnaire, we could check symptoms, e.g., heartburn when lying down or standing up, heartburn after meals, difficulty swallowing or pain with swallowing [20].

## 2. Materials and Methods

### 2.1. Study Group and Sample Collection

The patients were admitted for upper gastrointestinal endoscopy for various indications at the Department of Digestive Tract Diseases at the Barlicki Memorial Hospital in Lodz, Poland, from January 2019 to December 2020. The inclusion criteria encompassed a patient history of GERD, diagnosed based on typical symptoms and upper GI tract endoscopy. Patients with any other inflammatory disease of the gastrointestinal tract, Barrett’s esophagus, or gastric or esophageal neoplasia were excluded from this study. In total, 53 patients with GERD and 9 sex- and age-matched healthy controls were enrolled in this study. Among the GERD patients, 37 had non-erosive reflux disease (NERD) and 16 had erosive reflux disease (ERD). They were classified according to the Los Angeles classification as GERD grade A, B, C or D. Esophageal biopsies were collected from the lower part of the esophagus and kept at –80 °C for further analysis. This study was conducted in accordance with the ethical principles of the 1975 Declaration of Helsinki, and the independent Bioethics Committee of the Medical University of Lodz approved the study protocol (RNN/12/19/KE). All the participating subjects gave written, informed consent prior to enrollment.

### 2.2. RNA Isolation

RNA extraction was performed using the commercially available Total RNA Mini Kit (A&A Biotechnology, Gdynia, Poland) according to the manufacturer’s protocol. RNA purity and quantity were assessed spectrophotometrically using a Colibri Microvolume Spectrometer (Titertek Berthold, Colibri, Germany).

### 2.3. Real-Time PCR

cDNA synthesis was performed with the RevertAid First Strand cDNA Synthesis Kit (Fermentas, Burlington, Canada). A total of 1 µg of RNA was used in the reverse transcription reaction, with a final volume of 20 µL, following these incubation steps: 25 °C for 10 min, 50 °C for 15 min, 85 °C for 5 min and 4 °C for 10 min. mRNA expression quantification was carried out using real-time PCR with FAM dye-labeled TaqMan^®^ probes (Applied Biosystems, Waltham, MA, USA). The reaction mixture consisted of cDNA, TaqMan™ Gene Expression Master Mix, TaqMan™ Gene Expression Assays (*F2RL1:* Hs00608346_m1, *P2RX2:* Hs00247255_m1, *P2RX3:* Hs01125554_m1 and *P2RY2:* Hs00925146_m1) and RNase-free water, with a final volume of 10 μL. The cycling conditions were as follows: initial denaturation at 95 °C for 10 min, followed by 40 cycles of denaturation at 95 °C for 15 s and annealing/extension at 60 °C for 1 min. The obtained results were normalized to the expression of the hypoxanthine phosphoribosyltransferase 1 (*HPRT1*) gene (Hs02800695_m1) as an endogenous control. All experiments were performed in triplicate. The reaction was conducted using the LightCycler^®^ 96 Instrument (Roche, Basel, Switzerland). The initial amount of the template was evaluated using the Ct (cycle threshold) parameter. The Ct value represented the cycle number at which PCR amplification crossed a significant threshold. The relative expression level was calculated as 2^−∆Ct^ × 1000.

### 2.4. Dilated Intracellular Spaces

The DIS score was evaluated during routine microscopic examination of esophageal sections. Esophageal specimens were fixed in 10% neutral-buffered formalin at 4 °C for 24 h. After subsequent dehydration in sucrose, they were embedded in paraffin, sectioned into 5 µm slices and mounted onto slides. Sections were then stained with hematoxylin and eosin and examined using an Olympus CX43 microscope (Tokyo, Japan). The severity of DIS was assessed in one high-power field as follows: 0 (absent; ≤5 small intercellular spaces), 1 (≥6 small intercellular spaces and ≤5 large intercellular spaces) or 2 (≥6 large intercellular spaces), where “small” was defined as narrower than one lymphocyte in diameter, and “large” was defined as equal to or wider than one lymphocyte in diameter. DIS near the periphery of a biopsy was considered artifactual and was disregarded in this evaluation.

### 2.5. Health-Related Quality of Life

The Gastroesophageal Reflux Disease-Health-Related Quality of Life (GERD-HRQL) questionnaire was used to assess the severity of typical GERD symptoms. This tool is among the most frequently used symptom severity scales and is recommended by the European Association for Endoscopic Surgery. The best possible total GERD-HRQL score is 0 (asymptomatic for all items), while the worst possible score is 50 (incapacitated in all items). As the total GERD-HRQL score includes 51 possible values, it offers a high level of precision [20].

### 2.6. Statistical Analyses

Statistical analysis was performed using GraphPad Prism 8.0 (GraphPad Software Inc., La Jolla, CA, USA). Assumption of the normal distribution of differences was verified with the use of the Shapiro–Wilk test. As the normality assumption was violated, the significance of differences was tested with Mann–Whitney’s U test to compare two independent groups. For a multiple comparison, the Kruskal–Wallis test was applied. The data are expressed as a median with interquartile range. Analysis of the correlation between *F2RL1*, *P2RX2*, *P2RX3* and *P2RY2* expression and DIS or HRQL score was conducted by calculation of Spearman’s rank correlation coefficient. A heatmap showing the relation between the relative expression of *F2RL1*, *P2RX2*, *P2RX3* and *P2RY2* as well as DIS score is presented in mean values. Outliers were counted using the ROUT method and excluded. *p*-values < 0.05 were considered statistically significant.

## 3. Results

### 3.1. The Expression of F2RL, P2RX2, P2RX3 and P2RY2 in Patients with GERD

**Figure 1:**
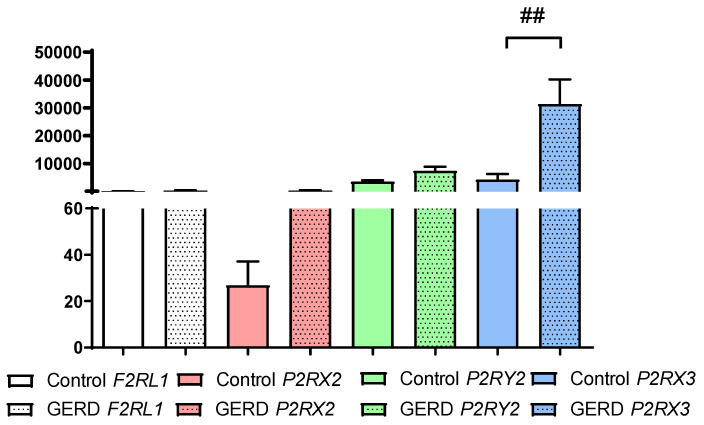
The expression of *F2RL1*, *P2RX2*, *P2RX3* and *P2RY2* in healthy controls (*n* = 9) and patients with GERD (*n* = 53). The Mann–Whitney test was used to compare the values; ## *p* < 0.01 vs. respective control.

As shown in Figure 1, F2RL1, P2RX2, P2RX3 and P2RY2 expression was detected in the esophagus of both healthy controls and patients with GERD. Overall, the relative expression of F2RL1 (77.60 ± 28.50 vs. 284.60 ± 67.72), P2RX2 (26.81 ± 10.27 vs. 274.40 ± 77.46) and P2RY2 (3476.00 ± 508.20 vs. 7215.00 ± 1338.00) was higher in the esophageal tissue of GERD patients than in healthy controls, although the differences were not statistically significant. Our real-time PCR analysis demonstrated a significantly higher expression of P2RX3 (4268 ± 2012 vs. 31,353 ± 8815, *p* < 0.01) in the esophagus of GERD patients compared to healthy controls (Figure 1). Notably, the relative expression of P2RX3 was markedly higher than that of F2RL1, P2RX2 and P2RY2 in the esophageal mucosa of GERD patients.

### 3.2. The Expression of F2RL1, P2RX2, P2RX3 and P2RY2 in NERD or ERD Type of GERD Patients

**Figure 2:**
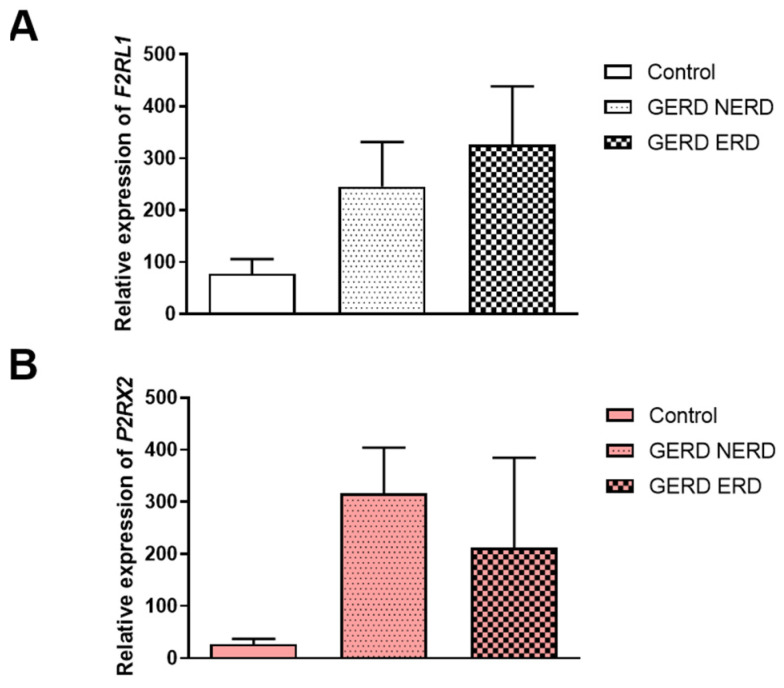

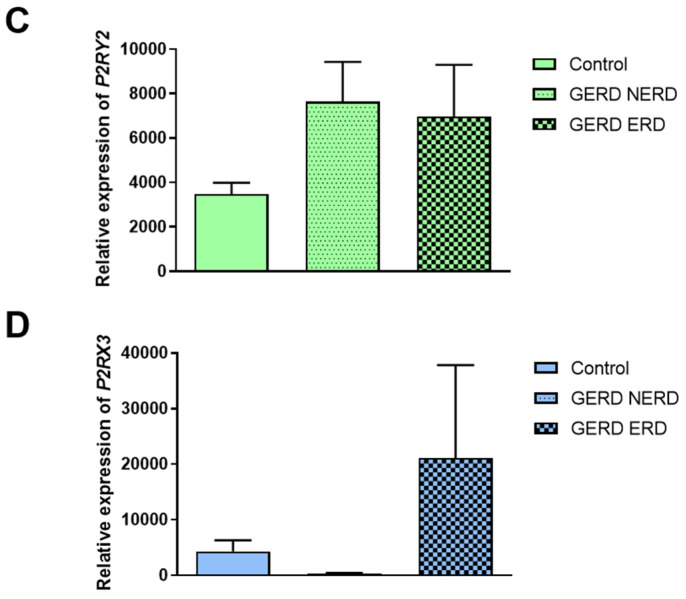
The expression of *F2RL1* (**A**), *P2RX2* (**B**), *P2RX3* (**C**) and *P2RY2* (**D**) in healthy controls (*n* = 9) and GERD patients with NERD (*n* = 32–36) or ERD (*n* = 12–15). The Kruskal–Wallis test was used to compare the values.

Division of GERD patients into non-erosive and erosive types of GERD revealed that the expression of *F2RL1* was non-significantly different in ERD compared to NERD as well as healthy controls (326.10 ± 112.30 vs. 266.90 ± 84.76 vs. 77.60 ± 28.50, Figure 2A). According to Figure 2B,C, non-significantly higher expression of *P2RX2* (302.20 ± 82.94 vs. 40.18 ± 17.78 vs. 26.81 ± 10.27) and *P2RY2* (7321.00 ± 1651.00 vs. 5306.0 ± 1738.00 vs. 3476.00 ± 508.0) in the esophageal mucosa obtained from NERD and ERD patients compared to healthy controls was observed. Of note, a lack of differences between the expression of *P2RX2* and *P2RY2* in NERD compared to ERD was documented. In patients with NERD, the expression of *P2RX3* in the esophageal mucosa was similar to that in healthy controls (Figure 2D).

### 3.3. The Association Between F2RL1, P2RX2, P2RX3 and P2RY2 Expression and DIS Score in Patients with GERD

**Figure 3:**
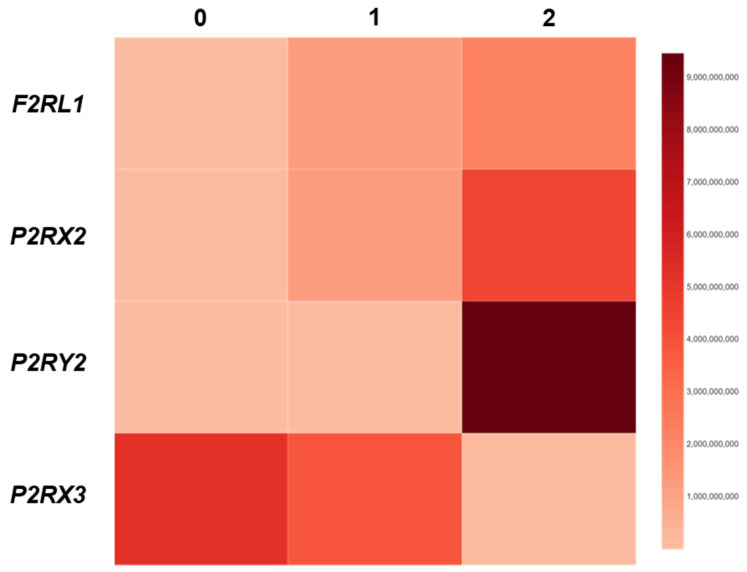
Heatmap showing the relation between the expression of *F2RL1*, *P2RX2*, *P2RX3* and *P2RY2* and DIS score (*n* = 24–30). The Spearman rank correlation test was used to analyze the association between the expression of genes and DIS score vs. respective control.

To investigate the association between the expression of *F2RL1*, *P2RX2*, *P2RX3* and *P2RY2* and the score of epithelial damage, the correlation coefficient between the expression levels of these genes and the DIS score was calculated. As shown in Figure 3, we observed that the expression of F2RL1 (r = 0.49; *p* < 0.05) and P2RX2 (r = 0.51; *p* < 0.01) correlated positively with the DIS score. Similarly, in GERD patients, the expression of P2RY2 (r = 0.60; *p* < 0.05) showed a positive correlation with the DIS score. The strongest correlation was observed between P2RY2 expression and the DIS score. On the other hand, in GERD patients, the expression of P2RX3 (r = −0.52; *p* < 0.05) correlated negatively with the DIS score (Figure 3).

### 3.4. The Association Between F2RL1, P2RX2, P2RX3 and P2RY2 Expression and HRQL of GERD Patients

**Figure 4:**
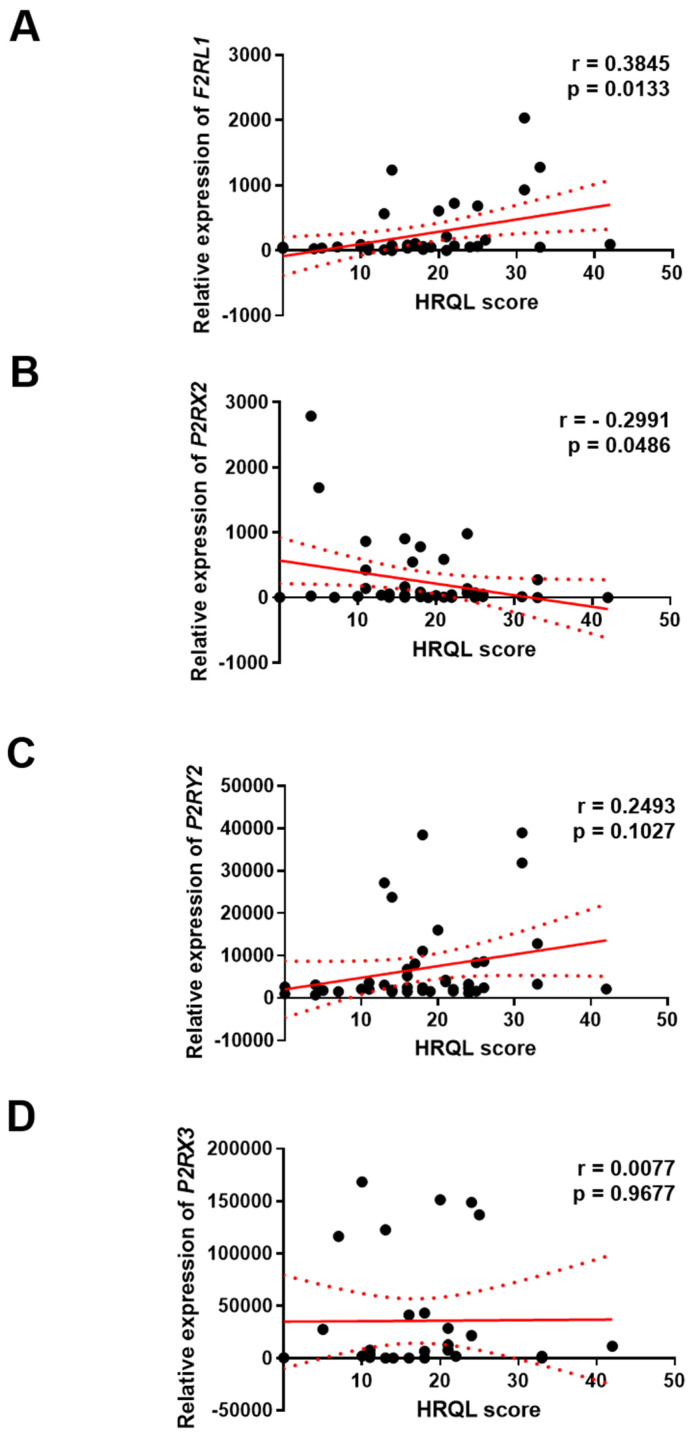
Correlations between the expression of *F2RL1* (**A**), *P2RX2* (**B**), *P2RX3* (**C**) and *P2RY2* (**D**) and HRQL in patients with GERD. The Spearman rank test was used to analyze the association between the expression of genes and GERD-HRQL vs. respective control.

To explore the clinical significance of *F2RL1*, *P2RX2*, *P2RX3* and *P2RY2* expression in GERD, the correlation between the level of *F2RL1*, *P2RX2*, *P2RX3* and *P2RY2* genes and the GERD-HRQL questionnaire score was investigated. We found a positive correlation between the level of *F2RL1* (r = 0.3845; *p* < 0.01) or *P2RY2* (and r = 0.2493; *p* > 0.05) genes and the HRQL score in GERD patients, respectively. On the other hand, a negative correlation between the level of the *P2RX2* gene and the HRQL score (r = −0.2991; *p* < 0.05) was documented. Of note, the level of the *P2RX3* gene seems to be not associated with the HRQL score (r = 0.0077; *p* > 0.05) in this group (Figure 4).

## 4. Discussion

Diagnosis, effective treatment and therapy monitoring of GERD patients, especially patients with NERD, are challenging, and novel approaches are needed to improve treatment strategies [6]. Protease-activated receptors and purinergic receptors mediate numerous cellular processes crucial for proper cell homeostasis, proliferation, differentiation or communication [4,5,7]. Here, we evaluated the expression of selected protease-activated and purinergic receptors in the esophagus of GERD patients in the context of its clinical significance.

In our study, we noted that PAR-2 (*F2RL1*) is overexpressed in the esophagus of NERD or ERD patients compared to healthy controls. Our findings are in accordance with the results presented by Kim et al., who noted a higher expression of PAR_2_ in GERD patients and its overexpression in the esophagus of patients with esophageal reflux symptoms [21]. Similarly, in our study, the expression of *F2RL1* is positively correlated with patients’ symptoms according to GERD-HRQL scores.

In line with this, some studies documented overexpression of PAR_2_ in the esophagus obtained from NERD and ERD patients compared to healthy controls [8,22,23]. Nevertheless, the clinical implication of PAR_2_ has not been clearly elucidated yet. In previous studies, mechanisms by which PAR_2_ may participate in GERD exacerbation were pointed out. Wulamu et al. noted that stress-induced inflammation in the esophagus of mice is accompanied by PAR_2_ overexpression [24]. In vitro studies documented that, in human esophageal epithelial cells affected by reflux with trypsin, upregulation of PAR_2_ expression is observed in a time- and dose-dependent manner, suggesting a crucial role for PAR_2_ in the inflammation related to GERD [5,25]. In fact, previous studies found that not only acid exposure but also PAR_2_ activation is needed to increase secretion of IL-8 [26]. It is worth noting that Shan et al. suggested that trypsin and PAR_2_ action may be directly responsible for the development of refractory GERD in patients under proton-pump inhibitor therapy [27]. However, further experimental studies employing in vitro and in vivo approaches are needed to explore the clinical potential of PAR_2_ expression.

Interestingly, in our study, the expression of PAR-2 is positively correlated to the severity of microscopic damage assessed by DIS. Our results suggest that PAR_2_ may be a promising clinical marker for monitoring epithelial permeability in GERD. DIS represents an impaired epithelial barrier which enables refluxate contents to access nerves endings and stimulate nociceptors [3,19]. An association has been found between DIS and exposure to acid, pepsin and bile [18,28]. It appears to be associated with symptoms of reflux and could be responsible for the symptoms of NERD.

The importance of DIS as an early marker of GERD has been studied since DIS has been shown to be produced by acidic and weakly acidic contents in the esophagus of healthy humans [29]. Caviglia et al. found that DIS was increased not only in patients with NERD but also in patients with heartburn symptoms but normal esophageal acid exposure [30]. The authors suspect a causative role of DIS in the development of reflux symptoms [28]. This raises hopes for the treatment of symptoms aimed at reducing DIS.

The significance of purinergic receptors in the pathogenesis of GERD is unknown. In GERD, the most explored purinergic receptors are P2RX2 and P2RX3 agonists, which, according to experimental studies, act as a regulator of the mechanosensory function of esophageal afferents [16]. An observational study where Shieh et al. evaluated numerous purinergic receptors documented that *P2RX3* and *P2RX7,* but not *P2RX2*, *P2RY1*, *P2RY2*, *P2RY4*, *P2RY6* and *P2RY12,* are upregulated in ERD when compared to asymptomatic patients or healthy controls [31]. The expression of both purinergic receptors altered in GERD, i.e., *P2RX3* and *P2RX7*, is positively correlated to the expression of transient receptor potential vanilloid receptor 1 (TRPV1), nerve growth factor and glial-derived neurotrophic factor [31]. TRPV1 and the above-mentioned neurotrophic factors participate in the development of inflammatory-related hyperalgesia, suggesting that P2RX3 may mediate sensitization of the inflamed esophagus [32].

Here, we documented significantly higher expression of *P2RX3* in the esophagus of GERD patients compared to healthy controls. In line with the results presented by Shieh et al., we found *P2RX3* overexpression in ERD but not in NERD, compared to healthy controls [31]. In contrast, our results documented a negative correlation between the expression of *P2RX3* and the severity of microscopic damage in GERD. Interestingly, the expression of seems to be not corelated with HRQL score. Perhaps the activation of *P2RX3* requires greater mucosal damage than in NERD, which would explain not only the normal values in NERD but also the lack of correlation with DIS and symptoms assessed by HRQL-GERD.

The inflammation releases inflammatory mediators such as ATP, which also stimulates the *P2X3* receptors. Also, acid in the lumen increases the sensitivity of the *P2X3* receptors to ATP. In the noninflamed esophagus, little or no acid enters the cell, resulting in a normal expression of *P2X3* receptors. The increased number of P2X3 receptors in the inflamed esophagus could lead to increased sensitivity to non-noxious stimuli in the afferent nerves. This increased sensitivity could partly explain the generation of reflux symptoms and hypersensitivity seen in GERD patients [5].

We found a positive correlation between *P2RX2* or *P2RY2* and DIS score. Higher expression of the above-mentioned purinergic receptors in GERD and NERD as well as ERD when compared to healthy controls was observed. The subgroup of purinergic receptors are specifically activated by ATP [5]. Each of these receptors have a different affinity to ATP, which may explain the different results in the study groups.

The action and function of *P2RX2* in the esophagus is poorly understood, but our results highlighted negative correlation between *P2RX2* expression and HRQL score in GERD patients. In contrast, the expression of *P2RY2* is positively correlated with GERD symptoms assessed by GERD-HRQL score. This could be explained by the observation that P2Y receptors appear to be more involved in esophageal motility [33]. The blockade of P2Y receptors reduced the amplitude of contractions of LES [33]. A more thorough examination of these relationships raises hopes for future treatments.

## 5. Conclusions

We conclude that expression of selected protease-activated and purinergic receptors such as F2RL1, P2RX2, P2RX3 and P2RY2 may serve for both GERD progression and GERD patients’ quality of life monitoring. Higher PAR2 expression was found in erosive reflux disease and may be associated with a higher intensity of symptoms perceived by patients with GERD. As high PAR-2 expression scores were significantly correlated with histologic alterations and severity of symptoms, a major role of PAR2 in the pathogenesis of GERD can be concluded from this study. The relative expression of P2X2 and P2Y2 were increased particularly in NERD, where they could mediate sensitization of the esophagus. PAR2, P2X2 and P2Y2 could be future therapeutic targets for reflux symptoms and BE prophylaxis.

## Data Availability

The datasets generated during and/or analyzed during the current study are available from the corresponding author on reasonable request.
